# ECMO in COVID-19—prolonged therapy needed? A retrospective analysis of outcome and prognostic factors

**DOI:** 10.1177/0267659121995997

**Published:** 2021-02-20

**Authors:** Esther Dreier, Maximilian Valentin Malfertheiner, Thomas Dienemann, Christoph Fisser, Maik Foltan, Florian Geismann, Bernhard Graf, Dirk Lunz, Lars Siegfried Maier, Thomas Müller, Robert Offner, David Peterhoff, Alois Philipp, Bernd Salzberger, Barbara Schmidt, Barbara Sinner, Matthias Lubnow

**Affiliations:** 1 Department of Internal Medicine II, University Hospital Regensburg, Regensburg, Germany; 2 Department of Surgery, University Hospital Regensburg, Regensburg, Germany; 3 Department of Cardiothoracic Surgery, University Hospital Regensburg, Regensburg, Germany; 4 Department of Anaesthesiology, University Hospital Regensburg, Regensburg, Germany; 5 Institute of Clinical Chemistry and Laboratory Medicine, Transfusion Medicine, University Hospital Regensburg, Regensburg, Germany; 6 Institute of Medical Microbiology and Hygiene, University of Regensburg, Regensburg, Germany; 7 Department for Infection Control and Infectious Diseases, University Hospital Regensburg, Regensburg, Germany; 8 Institute of Clinical Microbiology and Hygiene, University Hospital Regensburg, Regensburg, Germany

**Keywords:** COVID-19, extracorporeal membrane oxygenation, ARDS, ECMO, prolonged, SARS-CoV-2

## Abstract

**Background:**

The role of venovenous extracorporeal membrane oxygenation (VV ECMO) in patients with COVID-19-induced acute respiratory distress syndrome (ARDS) still remains unclear. Our aim was to investigate the clinical course and outcome of those patients and to identify factors associated with the need for prolonged ECMO therapy.

**Methods:**

A retrospective single-center study on patients with VV ECMO for COVID-19-associated ARDS was performed. Baseline characteristics, ventilatory and ECMO parameters, and laboratory and virological results were evaluated over time. Six months follow-up was assessed.

**Results:**

Eleven of 16 patients (68.8%) survived to 6 months follow-up with four patients requiring short-term (<28 days) and seven requiring prolonged (⩾28 days) ECMO support. Lung compliance before ECMO was higher in the prolonged than in the short-term group (28.1 (28.8–32.1) ml/cmH_2_O vs 18.7 (17.7–25.0) ml/cmH_2_O, p = 0.030). Mechanical ventilation before ECMO was longer (19 (16–23) days vs 5 (5–9) days, p = 0.002) and SOFA score was higher (12.0 (10.5–17.0) vs 10.0 (9.0–10.0), p = 0.002) in non-survivors compared to survivors. Low viral load during the first days on ECMO tended to indicate worse outcomes. Seroconversion against SARS-CoV-2 occurred in all patients, but did not affect outcome.

**Conclusions:**

VV ECMO support for COVID-19-induced ARDS is justified if initiated early and at an experienced ECMO center. Prolonged ECMO therapy might be required in those patients. Although no relevant predictive factors for the duration of ECMO support were found, the decision to stop therapy should not be made dependent of the length of ECMO treatment.

## Introduction

On 11 March 2020, the World Health Organization (WHO)^1^ declared coronavirus disease 2019 (COVID-19) outbreak caused by severe acute respiratory syndrome coronavirus 2 (SARS-CoV-2) a global pandemic. With over 63 million confirmed cases and almost 1.5 million deaths as of December 2, 2020,^2^ COVID-19 continues to be a disease of serious international public health concern. While most infections with SARS-CoV-2 remain asymptomatic or mild,^3^ some patients develop severe clinical manifestations with acute respiratory failure requiring intensive care unit (ICU) admission and invasive ventilation.^4^ Acute respiratory distress syndrome (ARDS) as a complication of COVID-19 is associated with poor prognosis and higher mortality rates among COVID-19 patients.^5,6^

During recent viral outbreaks like the 2009 influenza A (H1N1) pandemic, extracorporeal membrane oxygenation (ECMO) was used as a rescue therapy for patients with severe ARDS. Despite limited data, studies have shown that the initiation of ECMO in those patients has the potential to facilitate lung-protective ventilation and to improve survival compared to individuals treated with conventional therapies.^7–10^

The use of ECMO in patients with COVID-19-associated ARDS may be a promising strategy if maximal conventional treatment fails to assure adequate oxygenation and ventilation.^11,12^ The role of ECMO therapy in this pandemic is still controversial with some studies showing beneficial effects of ECMO in critically ill patients with COVID-19,^13,14^ while ECMO therapy has also been associated with poor outcomes and high mortality.^15^ Some reports indicate that individuals with ARDS caused by SARS-CoV-2 might need prolonged ECMO support with the associated requirement of ICU capacities.^16,17^

Therefore, the purpose of this study was to investigate the clinical course and outcomes of patients supported with venovenous (VV) ECMO for COVID-19-induced ARDS. Furthermore, we aimed to identify characteristics associated with prolonged ECMO treatment.

## Methods

### Study design and patients

This retrospective single-center study was conducted at the intensive care units of the University Hospital Regensburg (UKR), a tertiary German ECMO center. The analysis includes all consecutive patients who were placed on VV ECMO for COVID-19-induced ARDS between March 25 and May 7, 2020. SARS-CoV-2 infection was confirmed by real-time reverse transcription polymerase chain reaction (RT-PCR) from respiratory samples. The study population was classified into two groups: non-survivors and survivors. Survival was defined as ECMO weaning and ICU discharge. Depending on the total duration of ECMO support, survivors were further divided into a short-term (<28 days) and a prolonged (⩾28 days) ECMO group.

Because of the non-interventional study design, the requirement of patient consent and the necessity of approval for publication was waived by the ethics committee of our center (approval number: 20-1854-101).

### Data collection and clinical outcomes

Data were prospectively collected and retrospectively analyzed. Information was obtained from electronic medical records, discharge summaries and the ECMO database of the University Hospital Regensburg which records relevant clinical information on all ECMO patients treated at our hospital.

The following data were recorded and analyzed: (1) demographics, clinical characteristics, and comorbidities, (2) ventilatory settings and laboratory results before, 2 hours, and daily after ECMO initiation, (3) ECMO-characteristics and daily ECMO-settings, (4) anti-SARS-CoV-2 immunoglobulin G (IgG) levels in serum samples and viral load of SARS-CoV-2 in serum and respiratory samples,^18^ and (5) durations of treatment phases, complications on ECMO, and outcome.

### Statistical analysis

Data are reported as frequency and percentage for categorical variables and as median with interquartile range (IQR) for continuous variables. To compare categorical data between two groups, Fisher’s exact test was performed. For analysis of continuous variables in independent samples, Mann-Whitney U test was applied. All statistical tests were two-sided and a p-value of <0.05 was considered statistically significant. Analyses were performed using SPSS Statistics version 25.0 (IBM, Armonk, NY, USA). Figures were created in Microsoft Excel for Office 365 (Microsoft, Redmond, WA, USA) or in R version 4.0.2 (R Core Team, Vienna, Austria).

## Results

### Baseline characteristics

Between March 25 and May 7, 2020, a total of 53 patients with confirmed SARS-CoV-2 infection were admitted to our intensive care units. Sixteen patients were placed on VV ECMO due to COVID-19-associated ARDS. Prone ventilation before ECMO initiation was used in 15 patients. Ten patients had ECMO initiated at a referral hospital and were then transferred to our center by air or ground transport while receiving ECMO treatment. Details on baseline and pre-ECMO characteristics of the study population are reported in Table 1.

**Table 1.** Patient baseline and pre-ECMO characteristics.

Variable	All patients (*n* = 16)				Survivors (*n* = 11)		
		Survivors (*n* = 11)	Non-survivors (*n* = 5)	p-value	ECMO <28 days (*n* = 4)	ECMO ⩾28 days (*n* = 7)	p-value
Age (years)	59 (51–65)	56 (51–64)	62 (50–68)	0.641	58 (40–64)	56 (51–65)	0.682
Sex (male), *n* (%)	13 (81.3)	9 (81.8)	4 (80.0)	1.000	2 (50)	7 (100)	0.109
Body mass index (kg/m^2^)	27.5 (24.7–32.6)	26.5 (24.5–29.3)	33.1 (26.0–34.9)	0.189	27.3 (20.9–30.5)	26.5 (24.5–29.3)	1.000
Comorbidities							
Chronic pulmonary disease, *n* (%)	2 (12.5)	1 (9.1)	1 (20.0)	1.000	1 (25.0)	0 (0.0)	0.364
Arterial hypertension, *n* (%)	6 (37.5)	4 (36.4)	2 (40.0)	1.000	1 (25.0)	3 (42.9)	1.000
Chronic kidney disease, *n* (%)	1 (6.3)	1 (9.1)	0 (0.0)	1.000	0 (0.0)	1 (14.3)	1.000
Diabetes mellitus, *n* (%)	3 (18.8)	1 (9.1)	2 (40.0)	0.214	0 (0.0)	1 (14.3)	1.000
Duration between onset of symptoms and ECMO (days)	18 (13–25)^a^	15 (13–20)^b^	27 (25–30)^c^	**0.009**	13 (12–15)	18 (14–22)^d^	0.105
CRRT	4 (25.0)	1 (9.1)	3 (60.0)	0.063	1 (25.0)	0 (0.0)	0.364
SOFA score	10.0 (9.0–12.0)	10.0 (9.0–10.0)	12.0 (10.5–17.0)	**0.039**	9.5 (9.0–12.3)	10.0 (9.0–10.0)	0.867
LIS	3.3 (3.0–3.7)	3.3 (3.3–3.7)	3.0 (3.0–3.5)	0.323	3.5 (3.3–3.7)	3.3 (3.0–3.7)	0.421
PRESERVE score	4.0 (3.0–5.8)	4.0 (3.0–5.0)	4.0 (2.5–6.0)	1.000	4.0 (1.8–6.3)	5.0 (3.0–5.0)	0.788
Mechanical ventilation							
Duration (days)	8 (5–18)	5 (5–9)	19 (16–23)	**0.002**	5 (2–7)	5 (5–13)	0.300
Compliance (ml/cmH_2_O)	25.9 (19.4–29.0)	27.0 (19.0–30.5)	21.7 (19.3–26.8)	0.307	18.7 (17.7–25.0)	28.1 (26.8–32.1)	**0.030**
FiO_2_ (%)	0.95 (0.76–1.00)	1.00 (0.80–1.00)	0.8 (0.60–1.00)	0.330	0.95 (0.79–1.00)	1.0 (0.80–1.00)	1.000
PEEP (cmH_2_O)	15.0 (13.3–15.8)	15.0 (14.0–16.0)	14.0 (11.9–15.5)	0.386	14.5 (11.0–19.5)	15.0 (14.0–16.0)	0.752
P_Peak_ (cmH_2_O)	34.0 (31.0–35.0)	34.0 (31.0–35.0)	35.0 (30.0–38.5)	0.588	34.5 (29.5–39.5)	34.0 (31.0–35.0)	0.652
Tidal volume (ml/kg)	5.7 (4.3–6.3)	6.1 (4.2–6.8)	5.2 (4.3–5.7)	0.282	4.9 (3.6–7.4)	6.1 (4.4–6.8)	0.382
Respiratory rate (bpm)	25 (23–25)	25 (22–25)	25 (20–27)	0.938	25 (24–33)	25 (22–25)	0.388
PaO_2_/FiO_2_	83 (60–109)	65 (58–98)	107 (84–156)	0.064	83 (62–97)	65 (53–109)	0.494
Arterial blood gas							
pH	7.29 (7.20–7.36)	7.33 (7.27–7.39)	7.18 (7.14–7.22)	**0.001**	7.36 (7.31–7.41)	7.32 (7.27–7.37)	0.336
PaO_2_ (mmHg)	68 (60–89)	65 (53–76)	87 (73–102)	0.084	68 (60–91)	65 (52–76)	0.503
PaCO_2_ (mmHg)	67 (59–82)	62 (51–74)	78 (67–92)	0.138	62 (51–71)	67 (51–87)	0.564
Laboratory values							
Hemoglobin (g/dl)	9.6 (8.2–10.9)	10.3 (9.3–11.0)	8.0 (7.8–8.3)	**<0.001**	10.7 (9.3–12.5)	10.0 (9.3–11.0)	0.748
Platelet count (×10^3^/µl)	335 (186–402)	318 (197–394)	352 (135–461)	1.000	315 (197–393)	318 (197–487)	0.927
White blood cells (×10^3^/µl)	9.5 (8.3–11.9)	9.4 (7.6–11.4)	10.1 (8.7–19.7)	0.364	10.5 (8.9–21.0)	8.7 (6.5–10.0)	0.182
aPTT (seconds)	42 (34–46)	35 (30–43)	50 (45–80)	**0.003**	35 (28–42)	38 (30–43)	0.894
INR	1.15 (1.10–1.20)	1.10 (1.00–1.20)	1.20 (1.15–1.35)	0.087	1.00 (0.93–1.15)	1.10 (1.10–1.20)	0.097
D-dimers (mg/l)	4.0 (2.3–16.0)	4.0 (2.0–22.0)	4.0 (2.5–7.0)	0.602	3.5 (2.3–17.5)	7.0 (2.0–23.0)	0.612
Lactate (mg/dl)	12.0 (9.5–14.5)	13.0 (11.0–15.0)	9.0 (8.5–12.0)	0.059	12.5 (11.3–13.0)	15.0 (11.0–15.0)	0.458
C-reactive protein (mg/l)	212 (114–291)	131 (59–292)	265 (184–321)	0.278	207 (114–322)	131 (44–253)	0.527
Interleukin 6 (pg/ml)	673 (151–750)	663 (94–739)	698 (176–1384)	0.743	466 (171–850)	683 (57–739)	0.788

ECMO: extracorporeal membrane oxygenation; CRRT: continuous renal replacement therapy; SOFA: sequential organ failure assessment; LIS: lung injury score; PRESERVE: predicting death for severe ARDS on VV ECMO; FiO_2_: fraction of inspired oxygen; PEEP: positive end-expiratory pressure; P_Peak_: peak-pressure; bpm: breaths per minute; PaO_2_: arterial partial pressure of oxygen; PaCO_2_: arterial partial pressure of carbon dioxide; aPTT: activated partial thromboplastin time; INR: international normalized ratio.

Data are presented as *n* (%) or median (interquartile range). Bold face indicates a p-value <0.05 which was considered statistically significant.

a *n* = 14.

b *n* = 10.

c *n* = 4.

d *n* = 6.

### ECMO-characteristics, ventilatory parameters, and laboratory results

Initial ECMO configuration and size of cannulas were similar between non-survivors and survivors as well as within the group of survivors, irrespectively of the total duration of ECMO treatment (Supplemental Table S1). Cannulas were inserted via Seldinger’s percutaneous technique. Vascular access was established via femoral-jugular approach in 15 of 16 patients (93.8%). One non-survivor was initially cannulated with a NovaPort twin dual-lumen cannula (24Fr) which was implanted into the femoral vein. In that patient, conversion to femoral-jugular access with a separate drainage and return cannula was necessary due to refractory hypoxemia 1 day after ECMO initiation. Because of biventricular failure, another non-survivor needed conversion to venoarterial (VA) ECMO on day 6. One survivor required a second course of ECMO treatment, due to worsening oxygenation, in which a bi-caval double-lumen cannula was introduced. During ECMO treatment, heparin or argatroban were administrated to maintain an activated partial thromboplastin time (aPTT) of 50–60 seconds. Additionally, from the fifth patient onwards, all patients on ECMO received prophylactic aspirin (100 mg per day).

**Figure 1.**
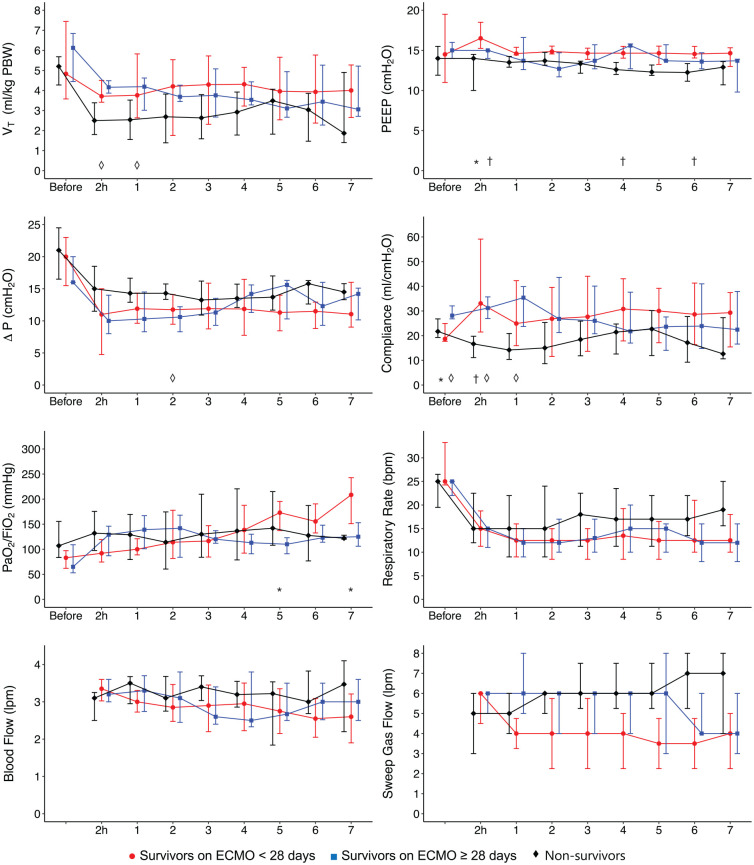
Levels of respiratory rate, positive end expiratory pressure (PEEP), driving pressure (∆P), compliance, PaO_2_/FiO_2_ ratio, tidal volume (*V*_T_), ECMO blood flow, and sweep gas flow over time. Days on ECMO except 2 hours = values 2 hours after ECMO implantation. A p-value < 0.05 was considered statistically significant for short-term group versus non-survivors (†), prolonged group versus non-survivors (◊), and short-term versus prolonged group (*). Data are shown as median and their interquartile range, and only for patients still on VV ECMO.

**Figure 2.**
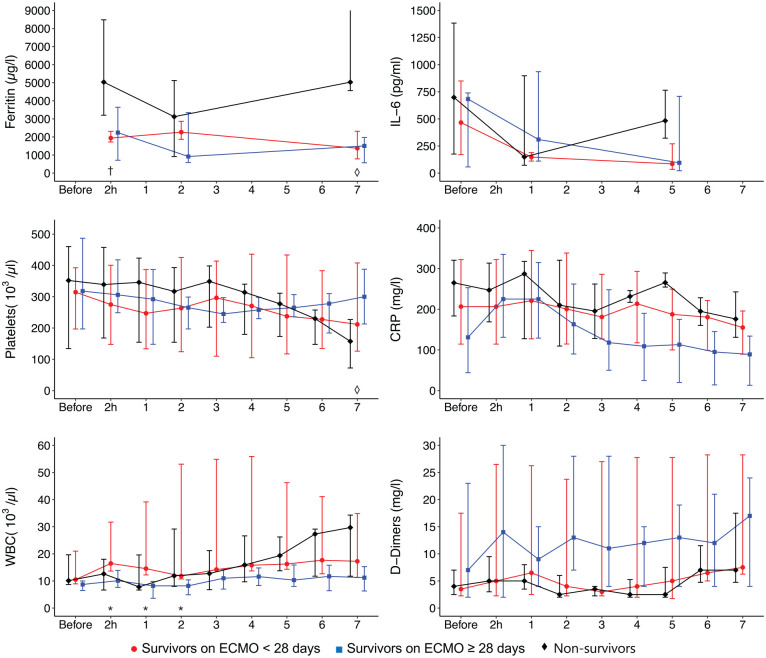
Levels of ferritin, interleukin 6 (IL-6), platelets, C-reactive protein (CRP), white blood cells (WBC), and D-dimers over time. Days on ECMO except 2 hours = values 2 hours after ECMO implantation. A p-value < 0.05 was considered statistically significant for short-term group versus non-survivors (†), prolonged group versus non-survivors (◊), and short-term versus prolonged group (*). Data are shown as median and their interquartile range, and only for patients tested at the respective time point.

Figure 1 shows changes in ECMO settings and ventilatory parameters within the first 7 days of ECMO therapy for survivors with short-term support, survivors with prolonged support, and non-survivors. The individual courses of lung compliance, PaO_2_/FiO_2_ ratio, ECMO blood flow, and sweep gas flow for each patient are demonstrated in the Supplemental Figure S1. Figure 2 shows selected laboratory results for the first 7 days on ECMO for non-survivors and survivors of the short-term as well as the prolonged ECMO group.

### Viral load and immune response

**Figure 3.**
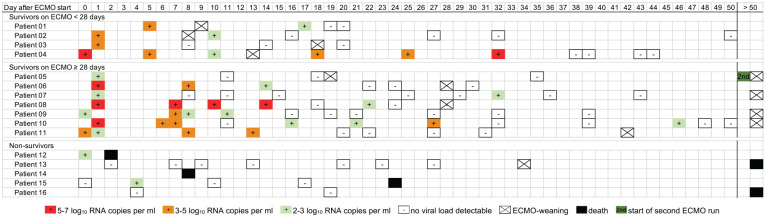
Timeline of the viral load of SARS-CoV-2 in respiratory samples for survivors on ECMO <28 days (*n* = 4), survivors on ECMO ⩾28 days (*n* = 7), and non-survivors (*n* = 5).

Within 7 days following ECMO implantation, viral load of SARS-CoV-2 in respiratory samples ranged from 4.36 to 6.60 log_10_ RNA copies per ml in survivors with short-term ECMO support and from 2.48 to 6.58 log_10_ RNA copies per ml in survivors with prolonged support. Viral RNA was no longer detectable or was 2.48 log_10_ RNA copies per ml in respiratory samples of non-survivors during the first week of ECMO therapy. Details on the kinetics of viral load in the respiratory tract over time are given in Figure 3.

**Figure 4.**
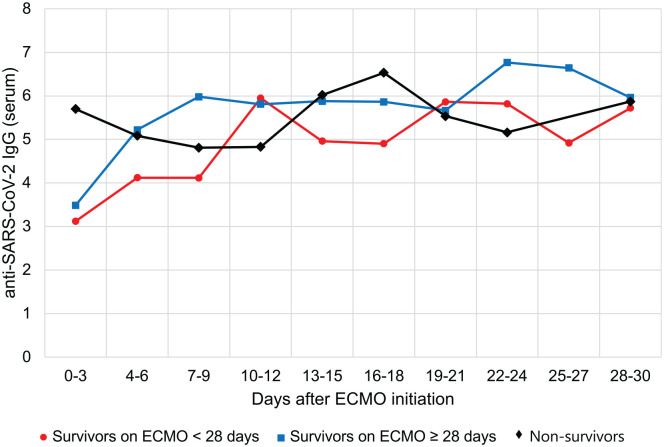
Levels of anti-SARS-CoV-2 IgG, presented as signal-to-cutoff ratio, in serum over time. Values ⩾1.0 were considered positive. Data are shown as median, and only for patients tested at the respective time point.

After ECMO initiation, SARS-CoV-2 RNA in blood samples was detected in one survivor with short-term ECMO support and in three patients receiving prolonged ECMO treatment. Eleven patients (68.8%) received COVID-19 convalescent plasma: three non-survivors, two survivors on ECMO < 28 days, and six survivors on ECMO ⩾ 28 days. No significant differences regarding the frequency of convalescent plasma therapy were observed between non-survivors and survivors or within the group of survivors, irrespectively of the length of ECMO support. IgG seroconversion against SARS-CoV-2 occurred in all 16 patients and antibody levels over time did not differ significantly between the groups (Figure 4). Further information on convalescent plasma therapy and the dynamics of IgG levels are provided in the Supplemental Figure S2. In one non-survivor, antibodies against SARS-CoV-2 were no longer detectable on day 5 of ECMO despite a positive test on day 2. In the autopsy of this patient, SARS-CoV-2-RNA was detected in the heart, lungs, liver, bowel, kidneys, and brain.

### Complications on ECMO and outcome

Out of the 16 patients receiving VV ECMO for COVID-19-associated ARDS, 12 (75.0%) were weaned from ECMO and 11 (68.8%) survived to ICU discharge and to the 6 months follow-up. Of the five non-survivors, four (80.0%) deceased on ECMO (three due to multiorgan failure, one due to terminal respiratory insufficiency without the option for lung transplantation) and one died after ECMO weaning due to CMV (cytomegalovirus) colitis refractory to antiviral therapy. Median time on ECMO was 24 (IQR: 5–74) days for non-survivors and 28 (IQR: 13–64) days for survivors (p = 0.529). Seven patients received prolonged ECMO support and four received short-term ECMO support with a median duration of 62 (IQR: 28–66) and 11 (IQR: 8–17) days (p = 0.006), respectively. As expected, length of ICU stay was significantly longer in the prolonged ECMO group (94 (IQR: 58–122) days) than in the short-term group (42 (IQR: 38–50) days, p = 0.012).

Frequencies of most complications on ECMO did not differ significantly between the groups. However, acute liver failure occurred in four of five non-survivors (80%), but in none of the survivors (p = 0.003). Lung complications including pneumothorax (one patient in the prolonged ECMO group, one in the short-term ECMO group) and pulmonary embolism (one non-survivor, four in the prolonged ECMO group) were observed in seven patients (43.8%). Two non-survivors suffered from gastrointestinal ischemia and one survivor in the prolonged ECMO group experienced gastrointestinal bleeding. Intracranial hemorrhage occurred in one patient with prolonged ECMO support. This patient had a good neurological outcome with a cerebral performance category (CPC) scale of one.

Nine of 16 patients (56.3%) required at least one oxygenator replacement with the first exchange after 3 (IQR: 2–11) days. In five of those patients, replacement was necessary within the first 3 days on ECMO. Compared to the short-term ECMO group, significantly more patients in the prolonged group required at least one new oxygenator (p = 0.024). Median number of oxygenator exchanges per 100 days of ECMO treatment was zero (IQR: 0.0–2.2), zero (IQR: 0.0–5.8), and 3.6 (IQR: 1.6–7.1) in the group of non-survivors, the short-term and the prolonged ECMO group, respectively (Supplemental Table S1). Seven patients (43.8%) did not need any replacement during their ECMO treatment with a median time on ECMO of 9 (8–24) days. The longest ECMO run without an oxygenator exchange was 43 days.

As of November 13, 2020, all survivors were discharged from hospital and alive. In consideration of the patients’ complications and short-term follow-up, it can be estimated that all patients will get back to a good neurological outcome (CPC 1–2) within the next months.

## Discussion

This work provides important information on the use of ECMO in COVID-19-induced ARDS and the clinical course of those patients. The most relevant findings of our study are: (I) Patients with COVID-19-induced ARDS need prolonged ECMO support which is not associated with poor outcomes. (II) Before ECMO initiation and within the first days of ECMO therapy, no relevant predictive differences in laboratory results and ventilatory or ECMO parameters were found between the short-term and the prolonged ECMO group. (III) Prolonged mechanical ventilation before ECMO and low viral load of SARS-CoV-2 in respiratory samples during the first days of ECMO support can be indicators for worse outcomes. (IV) While IgG seroconversion against SARS-CoV-2 occurred in all patients in our study population, we could not see an impact on the course of the disease or patient outcome.

Some studies have outlined that critically ill COVID-19 patients might require longer ECMO runtimes than patients with conventional ARDS.^16,17^ Contrary to this, Falcoz et al.^14^ reported shorter durations of ECMO treatment in COVID-19 patients which they attributed to the pathophysiology of SARS-CoV-2-induced respiratory failure.^19^ So far, the longest reported VV ECMO run in a patient with COVID-19-associated ARDS was 72 days prior to lung transplantation.^20^ Our patient population shows that prolonged ECMO support is necessary in patients with severe ARDS caused by SARS-CoV-2. Prolonged ECMO allowed native lung recovery and there was no need for lung transplantation within the group of survivors. With a median treatment duration of 28 days in the group of survivors, time on ECMO was much longer compared to randomized controlled trials on conventional ARDS^21^ and studies on H1N1-related ARDS.^10,22^ Data on prolonged ECMO support not related to COVID-19 and on follow-up of patients treated with ECMO for more than 28 days is limited. A recent analysis of risk factors for complete recovery of adults after VV ECMO treatment for respiratory failure indicated that long-term ECMO (⩾2 weeks) had negative effects on complete recovery.^23^ In contrast, further studies showed promising outcomes after median ECMO durations between 34 and 39 days.^24–26^ In order to ascertain whether prolonged ECMO in COVID-19-associated ARDS is justified, we stratified the survivors into a short-term (<28 days) and a prolonged (⩾28 days) ECMO group. Comparison of the two groups did not show any differences in outcomes and complications except for the frequency of oxygenator replacements. ECMO is a resource-intensive therapy^27^ and particularly prolonged support might influence ICU capacities and human and financial resources^28^—things that might be limited in a pandemic situation. These issues are in the focus of highly relevant ethical discussions as ICU capacities are crucial in many areas during the ongoing pandemic.^29^ It is obvious that the probability of required oxygenator exchanges increases with the duration of ECMO treatment. Furthermore, Camboni et al.^30^ considered oxygenator failure rather as a consumption than as a complication. Since early studies reported a high risk of abnormal coagulation in COVID-19 patients,^31,32^ we aimed for slightly higher aPTT levels (50–60 seconds) and further combined aspirin with heparin or argatroban to reduce the risk of oxygenator thrombosis and material consumption without observing any major bleeding complications in this population. The issue of patient selection and adequate treatment becomes even more important during this pandemic.^33^ Studies have shown that survival rate for patients requiring ECMO support was higher if transferred to high-volume centers compared to low-volume centers.^34^ As out-of-center VV ECMO initiation and interhospital transfer in COVID-19 patients can be considered as safe if carried out by qualified staff,^35^ those patients should be treated at experienced ECMO centers.

We looked for potential factors that could indicate the need for prolonged ECMO support in severe COVID-19 cases. Similar to findings in a study on long-term (⩾3 weeks) VV ECMO for non-COVID-19 ARDS,^24^ we did not find any differences between the short-term and prolonged ECMO group in demographics. Also the clinical course before and within the first 7 days on ECMO was comparable in both groups. One difference we observed was that patients in the prolonged ECMO group had higher lung compliances prior to ECMO initiation. Gattinoni et al.^36,37^ described two different pathophysiologic phenotypes of COVID-19 pneumonia in their studies: type H with higher compliances and type L with lower compliances. Additionally, earlier studies assumed that different pathologies of lung damage could influence the duration of ECMO support.^38^ These clinical observations are strengthened by a study on patients who died from COVID-19 and whose lung tissues showed an increased frequency of endothelialitis and thrombosis.^39^ Severe endothelial injury could play a crucial role in the need for prolonged ECMO support as it might take more time to recover. After ECMO initiation, compliance in the two groups was similar. Considering the patients’ individual courses, compliance tended to need more time to increase in patients with prolonged support. Nonetheless, native lung recovery was achieved in those patients after several weeks of therapy. Therefore, lack of improvement of lung function in the first weeks of ECMO or even temporarily worsening should not be seen as an indication to stop treatment.

It has to be mentioned that most studies did not exclude non-survivors when comparing data on short-term and prolonged ECMO.^24–26^ This needs to be taken into consideration as those studies reported a significantly longer duration of mechanical ventilation prior to ECMO initiation in the prolonged groups, whereas in our study, duration was similar between the two groups. Moreover, in accordance with non-COVID-19 ARDS,^40,41^ our results have shown that prolonged pre-ECMO ventilation can be an indicator for higher mortality in COVID-19-associated ARDS. Especially during this pandemic, prolonged pre-ECMO ventilation (⩾7 days) was considered a contraindication for ECMO therapy in some institutions.^42^ In addition, no or low viral load in respiratory samples during the first days on ECMO appeared to indicate poor outcomes, too. In recent studies, SARS-CoV-2 RNA in respiratory samples peaked within 5 days after symptom onset and decreased afterwards.^43,44^ As non-survivors also had higher SOFA scores and duration between onset of symptoms and ECMO start was longer, we think that this group was in advanced stages of the disease when ECMO was introduced. Therefore, we assume that earlier ECMO initiation could improve patient outcome in this population and should be further investigated. Nevertheless, survival rate in the present study is much higher compared to earlier studies^15^ and is in consistence with results of the to date largest study on ECMO in COVID-19.^45^

Except for one study on convalescent plasma therapy,^46^ literature on immunological responses in COVID-19 patients treated with ECMO is rare. In the study by Madariaga et al.,^46^ the two patients receiving ECMO therapy had the highest antibody titers within the study population of ten critically ill patients. In the days after plasma transfusion, antibody levels in non-ECMO patients increased, whereas levels decreased in the two ECMO patients. The influence of seroconversion and antibody levels on the course of the disease remains poorly understood and controversial.^43,47–49^ In our study population, all patients developed antibodies against SARS-CoV-2. IgG levels after ECMO initiation did not differ between the three groups and therefore, might not have influenced the clinical course. However, as all of our patients have to be considered as critically ill, our findings do not refute results indicating that antibody levels vary between patients with different disease severities.^50^

This study has notable limitations that should be considered. First, it was conducted as a retrospective single-center study at a highly experienced ECMO center which limits the generalizability of the results. Second, only a limited number of patients were included and third, therapy before ECMO initiation might have differed within the study population as the majority of the patients was treated at different hospitals prior to transfer to our ICUs.

## Conclusion

VV ECMO support in patients suffering from COVID-19 induced ARDS has its justification and should be initiated early and at an experienced ECMO center. Prolonged ECMO therapy might be required in those patients. Although no relevant predictive factors for the duration of ECMO support were found, the decision to stop therapy should not be made in dependency of the length of ECMO treatment as good clinical outcomes can be reached even after prolonged ECMO treatment. However, the initiation of ECMO during this pandemic has to be discussed in a multidisciplinary team on a case by case basis and in consideration of the local situation and the availability of trained staff and resources.

## Supplemental Material

sj-pdf-1-prf-10.1177_0267659121995997 – Supplemental material for ECMO in COVID-19—prolonged therapy needed? A retrospective analysis of outcome and prognostic factors Click here for additional data file. Supplemental material, sj-pdf-1-prf-10.1177_0267659121995997 for ECMO in COVID-19—prolonged therapy needed? A retrospective analysis of outcome and prognostic factors by Esther Dreier, Maximilian Valentin Malfertheiner, Thomas Dienemann, Christoph Fisser, Maik Foltan, Florian Geismann, Bernhard Graf, Dirk Lunz, Lars Siegfried Maier, Thomas Müller, Robert Offner, David Peterhoff, Alois Philipp, Bernd Salzberger, Barbara Schmidt, Barbara Sinner and Matthias Lubnow in Perfusion
