# Supplementary material for: ECMO in COVID-19—prolonged therapy needed? A retrospective analysis of outcome and prognostic factors
Source: Perfusion. 2021 Feb 20;36(6):582–91. doi: 10.1177/0267659121995997 (PMC8369905; doi:10.1177/0267659121995997)

Supplemental Material

Table S1. ECMO Characteristics

Patient	Days on ECMO	Drainage Cannula		Return Cannula		Type of oxygenator	Number of oxygenators
		Vessel	Size (Fr)	Vessel	Size (Fr)		
**Survivors on ECMO < 28 days**							
01	9	Femoral vein	23	Jugular vein	17	Hiltie LT	1
02	8	Femoral vein	21	Jugular vein	17	ECC.O 5	1
03	18	Femoral vein	23	Jugular vein	19	Cardiohelp 7.0	1
04	13	Femoral vein	21	Jugular vein	17	Hiltie LT	2
**Survivors on ECMO ≥ 28 days**							
05 [a]	62	Femoral vein	21	Jugular vein	21	Hiltie LT	3
06	28	Femoral vein	23	Jugular vein	21	Cardiohelp 7.0	3
07	71	Femoral vein	21	Jugular vein	19	Nautilus	5
08	28	Femoral vein	23	Jugular vein	21	Hiltie LT	2
09	66	Femoral vein	21	Jugular vein	17	Nautilus	2
10	64	Femoral vein	23	Jugular vein	21	ECC.O 5	2
11	42	Femoral vein	23	Jugular vein	19	Hiltie LT	5
**Non-survivors**							
12	2	Femoral vein	19	Jugular vein	17	Hiltie LT	1
13	34	Femoral vein	21	Jugular vein	17	Hiltie LT	1
14	8	Femoral vein	21	Jugular vein	19	ECC.O 5	1
15	24	Femoral vein	23	Jugular vein	19	Cardiohelp 7.0	1
16	113	Femoral vein	24	Femoral vein (double-lumen)	24	ECC.O 5	6

[a] Patient required two ECMO runs (first run: 19 days, 2 oxygenators; second run: 43 days, bi-caval double-lumen cannula, 1 oxygenator (ECC.O 5))

Figure S1.

Individual time courses (days on ECMO except 2h = values two hours after ECMO implantation) of compliance, PaO_2/FiO_2 ratio, ECMO blood flow, and sweep gas flow.
One patient in the prolonged group was weaned on day 19, but required a second ECMO run which started on day 75 and lasted 43 days.

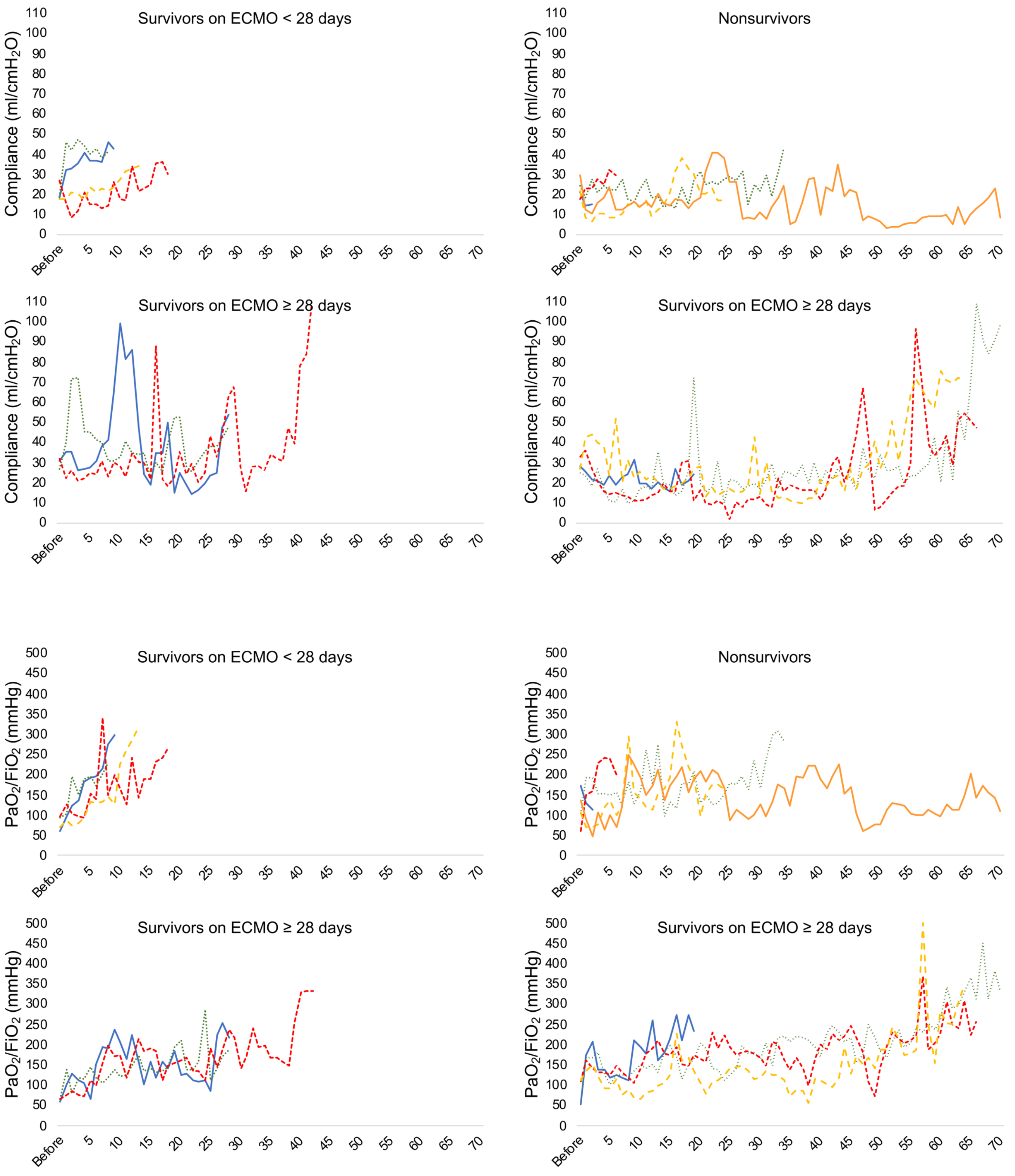

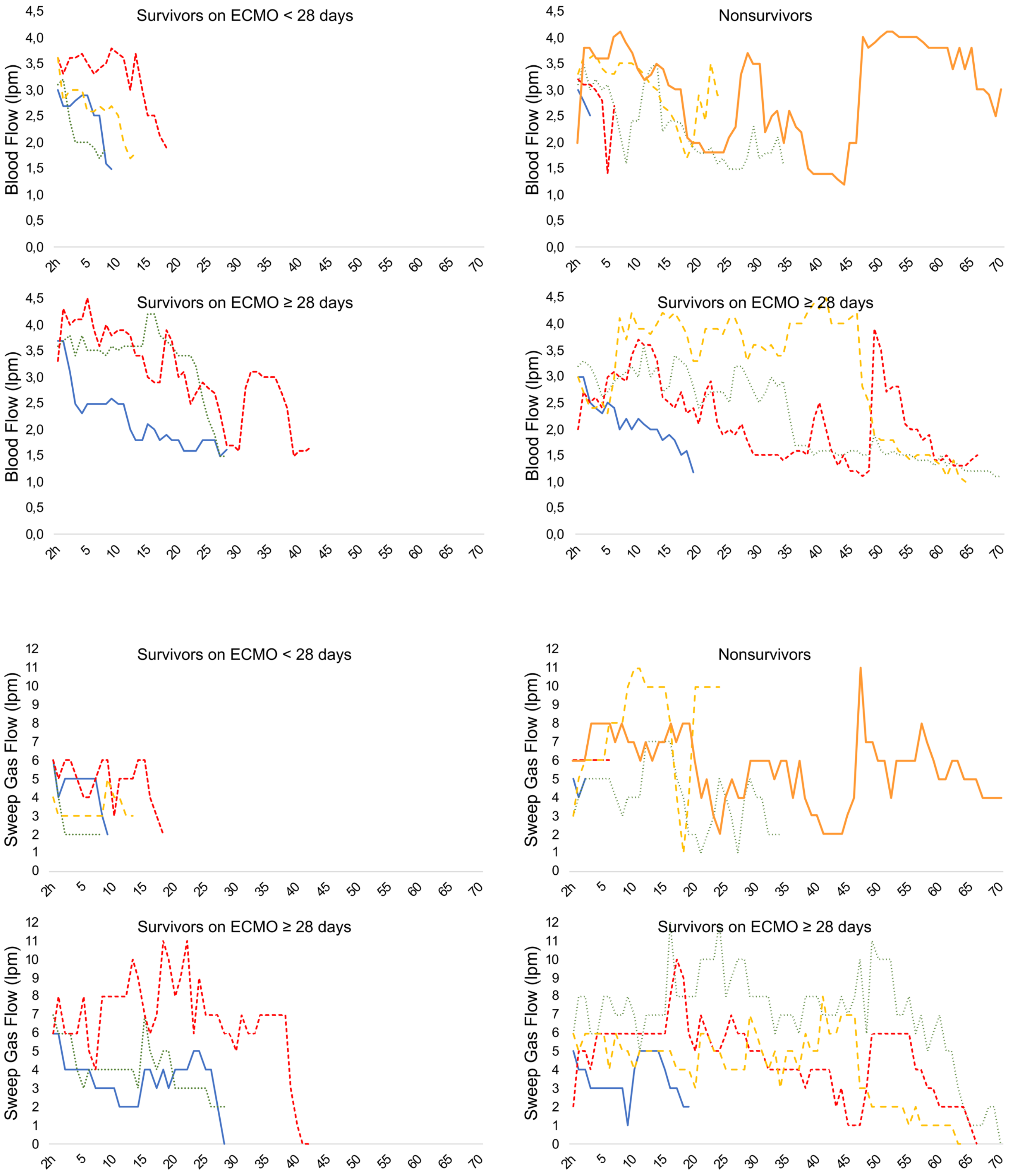

Survivors on ECMO < 28 days
Nonsurvivors
Survivors on ECMO ≥ 28 days
Survivors on ECMO ≥ 28 days
Blood Flow (lpm)
4,5
4,0
3,5
3,0
2,5
2,0
1,5
1,0
0,5
0,0
2h 5 10 15 20 25 30 35 40 45 50 55 60 65 70
Survivors on ECMO < 28 days
Nonsurvivors
Survivors on ECMO ≥ 28 days
Survivors on ECMO ≥ 28 days
Sweep Gas Flow (lpm)
12 11 10 9 8 7 6 5 4 3 2 1 0
2h 5 10 15 20 25 30 35 40 45 50 55 60 65 70

Figure S2.

Timeline of anti-SARS-CoV-2 IgG, presented as signal-to-cutoff ratio, in serum samples for patients on ECMO < 28 days (n=4), patients on ECMO ≥ 28 days (n=7), and non-survivors (n=5).

Values ≥ 1.0 were considered positive. Dashed line: ECMO-discontinuation; solid line: deceased (patient 13 died 47 days after ECMO weaning, patient 16 died on day 113 on ECMO); dotted line: start of second ECMO run; ▲: convalescent plasma therapy; ■: convalescent plasma therapy before ECMO start.

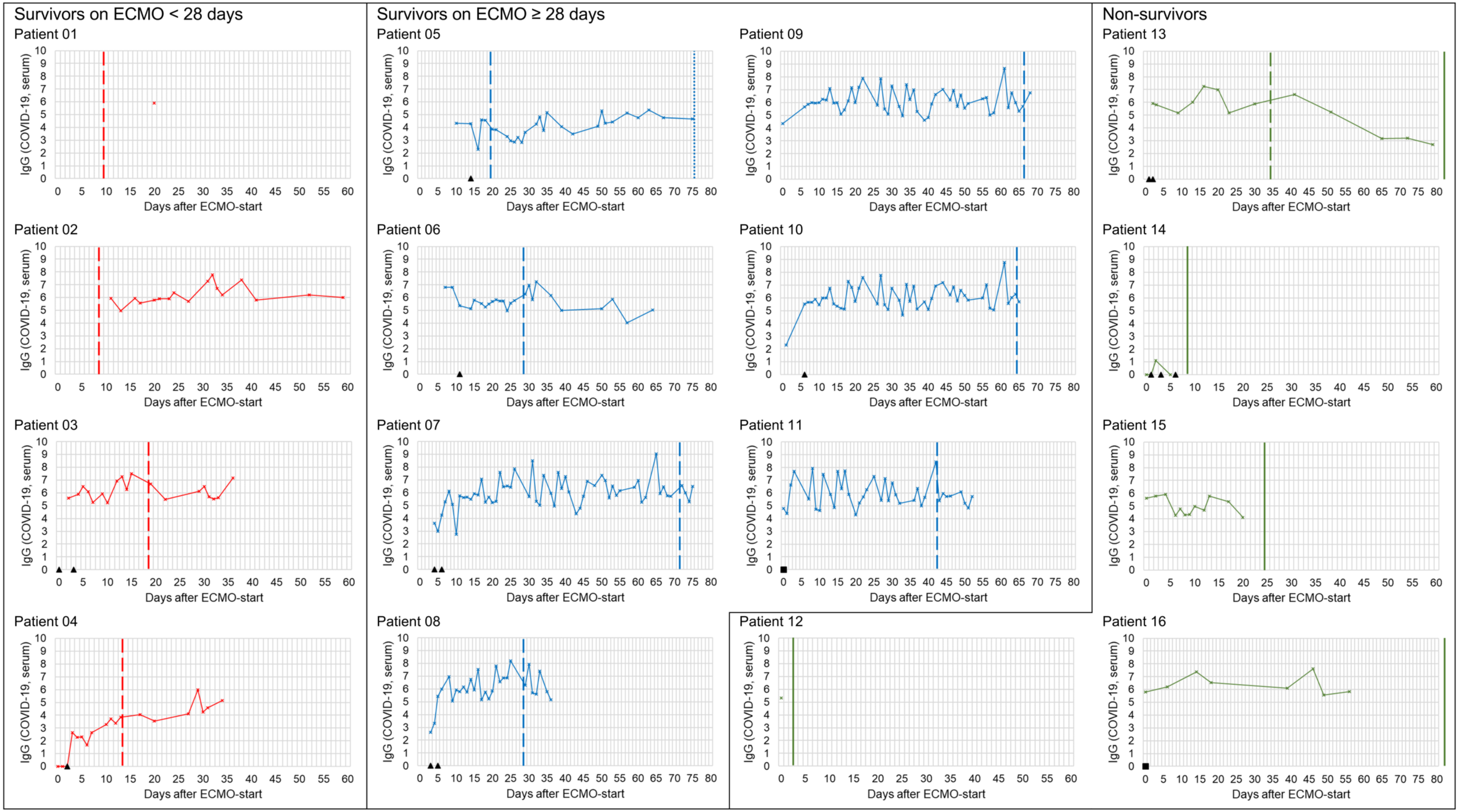

Supplement: sj-pdf-1-prf-10.1177_0267659121995997 – Supplemental material for ECMO in COVID-19—prolonged therapy needed? A retrospective analysis of outcome and prognostic factors [file sj-pdf-1-prf-10.1177_0267659121995997.pdf]
